# Short term outcome after left atrial appendage occlusion with the AMPLATZER Amulet and WATCHMAN device: results from the ORIGINAL registry (saxOnian RegIstry analyzinG and followINg left atrial Appendage cLosure)

**DOI:** 10.1186/s12872-022-02708-4

**Published:** 2022-06-16

**Authors:** Lucie Kretzler, Christoph Mues, Carsten Wunderlich, Anke Langbein, S. G. Spitzer, Ulrich Gerk, Sebastian Schellong, Thomas Ketteler, Hans Neuser, Marcus Schwefer, Ruth Strasser, Karim Ibrahim, Steffen Schoen, Marian Christoph

**Affiliations:** 1 Clinical Study Center (CSC), Berlin Institute of Health (BIH), Charité – Universitätsmedizin Berlin, Corporate Member of Freie Universität Berlin, Humboldt-Universität zu Berlin, Campus Virchow Klinikum, Augustenburger Platz 1, 13353 Berlin, Germany; 2 Dresden University of Technology, Dresden, Germany; 3 St.-Johannes-Hospital Dortmund, Dortmund, Germany; 4 Technische Universität Dresden, Pirna Hospital, Pirna, Germany; 5 Praxisklinik Herz und Gefäße Dresden, Dresden, Germany; 6 Städtisches Klinikum Dresden, Dresden, Germany; 7 HELIOS Klinikum Aue, Aue, Germany; 8 HELIOS Klinikum Plauen, Plauen, Germany; 9 Elblandklinikum Riesa, Riesa, Germany; 10 Klinikum Hochrhein, Waldshut-Tiengen, Germany; 11 Technische Universität Dresden, Campus Chemnitz , Klinikum Chemnitz, Flemmingstrasse 2, 09116 Chemnitz, Germany

**Keywords:** Left atrial appendage, Atrial fibrillation, Stroke prevention, Patient registry

## Abstract

**Background:**

Various randomized multicenter studies have shown that percutaneous left atrial appendage closure (LAAC) is not inferior in stroke prevention compared to vitamin K antagonists (VKA) and can be performed safely and effectively.

**Aims:**

The prospective multicenter ORIGINAL registry in the Free State of Saxony (saxOnian RegIstry analyzinG and followINg left atrial Appendage cLosure) investigated the efficiency and safety of LAAC with Watchman or Amulet device in a real word setting. A special focus was put on the influence of LAAC frequency on periprocedural efficiency and safety.

**Methods and results:**

The total of 482 consecutive patients (Abbott Amulet N = 93 and Boston Scientific Watchman N = 389) were included in the periinterventional analyses. After 6 weeks, 353 patients completed the first follow-up including transoesophageal echocardiography (TEE) (73.2%). Successful LAAC could be performed in more than 94%. The complication rate does not significantly differ between device types (*p* = 0.92) according to Fischer test and comprised 2.2% in the Amulet and 2.3% in the Watchman group. The kind of device and the frequency of LAAC per study center had no influence on the success and complication rates. Device related thrombus could be revealed more frequently in the Watchman group (4.5%) than in the Amulet group (1.4%) but this difference is still not significant in Fisher test (*p* = 0.14). Same conclusion can be made about residual leakage 1.1% versus 0% [not significant in Fisher test (*p* = 0.26)]. Dual antiplatelet therapy followed the intervention in 64% and 22% of patients were discharged under a combination of an anticoagulant (VKA/DOAC/Heparin) and one antiplatelet agent.

**Conclusions:**

The ORIGINAL registry supports the thesis from large, randomized trials that LAAC can be performed with a very high procedural success rate in the everyday clinical routine irrespective of the used LAA device (Watchman or Amulet). The postprocedural antithrombotic strategy differs widely among the participating centers.

*Trial registration* Name of the registry: "saxOnian RegIstry analyzinG and followINg left atrial Appendage cLosure", Trial registration number: DRKS00023803; Date of registration: 15/12/2020 'Retrospectively registered'; URL of trial registry record: https://www.drks.de/drks_web/navigate.do?navigationId=trial.HTML&TRIAL_ID=DRKS00023803.

## Introduction

Interventional left atrial appendage closure (LAAC) has advanced to a well-established alternative to oral anticoagulation for stroke prevention in patients with atrial fibrillation, especially in subjects who are not suitable for systemic anticoagulation [1, 2]. Various randomized multicenter studies have shown that LAAC is not inferior in stroke prevention compared to VKA and can be performed safely and effectively [3, 4]. Thus, the 2.3 Year follow-up data of the PROTECT AF study could demonstrate that the "local" strategy of left atrial appendage closure is noninferior to "systemic" anticoagulation with warfarin [4]. Furthermore, the subsequent PREVAIL trial showed a significantly improved procedural safety in comparison to the PROTECT AF data [3]. Based on these studies, the guidelines also support LAAC in patients who are not suitable for OAC. However due to the relatively small number of cases in the randomized trials, further clinical registries are desirable as additional confirmation of the randomized study data in everyday routine. Therefore, the current study aimed to evaluate the efficacy and safety of LAAC with the AMPLATZER Amulet and WATCHMAN Left Atrial Appendage Closure 2.5 generation device and focuses on the comparison of the two different devices regarding the periprocedural as well as short-term outcome data. These data were analysed with respect to the number of procedures/year/participating study center.

## Methods

### Study design

The ORIGINAL regIstry (saxOnian RegIstry analyzinG and followINg left atrial Appendage cLosures) represents a multicenter prospective clinical registry comprising nine hospitals in the Federal State of Saxony, Germany. The study was approved by the institutional ethical review board. All data were collected, managed and analysed at the Technische Universität Dresden and Berlin Institute of Health at Charité – Universitätsmedizin Berlin (ethics approval: University of Dresden: EK 245062014).

Trial registration: Name of the registry: "saxOnian RegIstry analyzinG and followINg left atrial Appendage cLosure", Trial registration number: DRKS00023803; Date of registration: 15/12/2020 'Retrospectively registered'; URL of trial registry record: https://www.drks.de/drks_web/navigate.do?navigationId=trial.HTML&TRIAL_ID=DRKS00023803

The *primary efficiency endpoint* of current analysis was the proportion of successful LAA closure in dependence of the used device (Amplatzer™ Amulet™ LAA Occluder; WATCHMAN Left Atrial Appendage Closure 2.5 generation device) and in dependence of the number of procedures performed annually per participating study center.

The *secondary efficiency endpoints* included total procedure time, the fluoroscopy time, contrast agent consumption during LAAC and the number of mismeasured discarded devices in dependence of the used device and in dependence of the number of procedures performed annually per study center. Furthermore, the proportion of devices with a residual leakage ≥ 5 mm 6 weeks after LAAC were recorded.

The *primary safety endpoint* of current analysis was the occurrence of periinterventional complications during LAAC (pericardial effusion, thromboembolic events, access complications, device dislocation, and death).

The *secondary safety endpoint* was the occurrence of device related complications 6 weeks after LAAC. Additionally, the regime of postinterventional antithrombotic therapy within the first 6 weeks after LAAC was recorded.

### Study population and protocol

Eligible subjects for the registry were consecutive male or female adults > 18 years of age suffering from atrial fibrillation or atypical atrial flutter (AF) and high thromboembolic risk [CHA2DS2-VASc ≥ 2 (male) ≥ 3 (female)] with an indication for LAAC according to the current ESC guidelines [1]. No other inclusion criteria were necessary. If the patient provided written consent for the registry, the clinical data, intraprocedural data and intrahospital complications were recorded. The LAAC were performed in 9 different hospitals in Saxony. The exact intraprocedural approach and the choice of the device were determined by the treating interventionalist. Randomization to a particular device group or statistical based matching with matched-pair analyses of both groups was not performed.

### Follow-up

Following the LAAC and 24 h after the procedure a neurological examination was conducted. Additionally, within 24 h after the procedure the access sites were clinically examined. In case of suspect local findings (hematoma, occurrence of new bruits, painful groin) a Doppler ultrasound examination of the access site was performed to rule out pseudo aneurysm or AV-fistula. Further, a transthoracic echocardiography to rule out device dislocation and pericardial effusion was carried out. In the case that the LAAC device could not be visualised with echocardiography, X-ray was performed.

6 weeks after LAAC a clinical follow-up including a TEE examination was performed. During this follow-up the occurrence of clinical complications as well as possible device dislocation, residual LAA leakage and device associated thrombi were recorded.

### Statistical analysis

The collected data were tested for normal distribution. Numeric variables were described by means and standard deviation (SD). Group comparison of continuous variables was assessed using two-sample Welch t-test. The total procedure duration, the fluoroscopy time, and the amount of contrast agent were subjected to the square root transform to stabilize their variance. Group comparison of binary variables were assessed using the Fisher exact test. The influence of individual variables to the lost-to-follow-up was assessed using binomial logistic regression and tested using χ^2^ likelihood ratio test. Multiple testing was corrected using the Bonferroni method and the significance level was set to *p* < 0.05/39 ≈ 0.0013.

## Results

### Study population

In total 482 consecutive patients were included in the periinterventional analysis. Both treatment groups (AMPLATZER Amulet N = 93 and WATCHMAN Left Atrial Appendage Closure 2.5 generation device N = 389) were balanced regarding the demographics and clinical baseline characteristics (Tables 1 and 2). After 6 weeks, 353 patients completed the 6-week follow-up including TEE (73.2%). A matched-pair analysis was not performed due to the small number of patients. The mean age of the treated patients was 74.41 (SD 8.775) in the Amulet group and 75.09 (SD 8.537) years in the Watchman group. Patients treated with the Amulet device had the mean CHA_2_DS_2_-VASc score of 4.22 (SD 1.5). The mean CHA_2_DS_2_-VASc score in the Watchman group was 4.04 (SD 1.5). The most predominant indication were previous bleeding complications with 70% in the Amulet group and 81% in the Watchman group. The detailed indications are listed in Table 1. The preinterventional anticoagulation varied widely, with DOAC monotherapy predominating (Amulet group: 48%; Watchman group 44%, detailed information is shown in Table 1).

**Table 1:** Baseline characteristics

	Amulet	Wachman	*p* value
*LAA closure*			
Total population	93	389	
Sex, male N (%)	56 (58%)	256 (66%)	0.310
Alter	74.41 ± 8.775	75.09 ± 8.537	0.500
CHA^2^DS^2^-VASc (Punkte)	4.22 ± 1.548	4.04 ± 1.546	0.334
HAS-BLED	3.34 ± 1.238	3.62 ± 1.105	0.051
LVEF (%)	55.62 ± 9.740	53.75 ± 10.491	0.101
LAA ostial diameter (mm)	17.97 ± 3.038	19.72 ± 3.463	0.105
AF paroxysmal	39 (45.3%)	171 (43.4%)	0.500
AF persistent	21 (19.8%)	70 (18.2%)	0.310
AF permanent	30 (28.3%)	148 (38.4%)	0.229
GFR prior to implantation	61.91 ± 26.834	59.01 ± 24.551	0.343
*Indication for LAA closure*			
Intracranial bleeding	26 (25%)	111 (29%)	0.533
Gastrointestinal bleeding	28 (26%)	128 (33%)	0.171
Urogenital bleeding	8 (08%)	23 (6%)	0.342
Other bleeding	12 (11%)	49 (13%)	0.789
High bleeding risk	1 (1%)	10 (3%)	0.386
Reccurent falls	1 (1%)	5 (1%)	0.870
Lack of compliance	1 (1%)	3 (1%)	0.772
Cognitive deficit	0 (00%)	5 (1%)	0.272
NSAID/Steroid intake	2 (2%)	0 (00%)	0.004
Hemodialysis	2 (2%)	4 (1%)	0.381
Other contraindication	10 (9%)	28 (7%)	0.644
Patient request	0 (00%)	10 (3%)	0.118
Left atrial appendage isolation	2 (02%)	2 (1%)	0.118
Intraatrial thrombi despite OAK	8 (8.6%)	7 (1.799%)	0.001
*Anticoagulation*			
ASA monotherapy	11 (11.3%)	34 (8.8%)	0.358
VKA monotherapy	12 (12.3%)	72 (18.7%)	0.183
DOAK monotherapy	50 (48.1%)	170 (44.2%)	0.080
LMWH/Fondaparinux monotherapy	6 (8.5%)	41 (10.1%)	0.233
Dual antiplatelet	1 (1.9%)	11 (2.6%)	0.330
VKA + antiplatelet	0 (0.0%)	9 (2.3%)	0.139
DOAK + antiplatelet	7 (6.6%)	11 (2.9%)	0.032
LMWH + antiplatelet	1 (0.9%)	3 (0.8%)	0.396
Triple therapy	0 (0.0%)	8 (2.1%)	0.163
No anticoagulation	6 (6.6%)	26 (6.8%)	0.936
*Co morbidities*			
Ischemic stroke or TIA	25	117	0.534
Diabetes mellitus	34	165	0.303
Vascular Disease	25	88	0.037
Hypertension	88	369	0.849
Congestive heart failure	11	95	0.027

**Table 2:** baseline characteristics follow up in 6 weeks

	Amulet	Watchman	*p* value
*LAA closure*			
Total population	76	277	
Sex, male N (%)	43 (56.6%)	233 (84.1%)	0.472
Alter	74.8 ± 7.9	74.9 ± 8.7	0.999
CHA^2^DS^2^-VASc (Punkte)	4.2 ± 1.5	4.2 ± 1.6	0.994
HAS-BLED	3.4 ± 1.3	3.6 ± 1.1	0.946
LVEF (%)	55.1 ± 10.7	53.6 ± 10.4	0.996
LAA ostial diameter (mm)	17.6 ± 2.7	19.8 ± 3.4	0.937
AF paroxysmal	32 (42.1%)	150 (43.5%)	0.983
AF persistent	18 (23.7%)	62 (18.0%)	0.886
AF permanent	25 (32.9%)	133 (38.6%)	0.926
*Indication for LAA closure*			
Intracranial bleeding	20 (26.3%)	96 (27.8%)	0.977
Gastrointestinal bleeding	15 (19.7%)	117 (33.9%)	0.797
Urogenital bleeding	7 (9.2%)	20 (5.8%)	0.826
Other bleeding	11 (14.5%)	46 (13.3%)	0.970
High bleeding risk	1 (1.3%)	8 (2.3%)	0.853
Reccurent falls	1 (1.3%)	4 (1.2%)	0.959
Lack of compliance	1 (1.3%)	3 (0.9%)	0.855
Cognitive deficit	0 (0.0%)	5 (1.4%)	0.634
NSAID/Steroid intake	2 (2.6%)	0 (0.0%)	0.027
Hemodialysis	1 (1.3%)	4 (1.2%)	0.959
Other contraindication	15 (19.7%)	30 (8.7%)	0.844
Patient request	0 (0.0%)	10 (2.9%)	0.629
Left atrial appendage isolation	1 (1.3%)	2 (0.6%)	0.688
*Anticoagulation*			
No anticoagulation	0 (0.0%)	0 (0.0%)	–
ASA monotherapy	3 (3.9%)	20 (5.8%)	0.890
VKA monotherapy	8 (10.5%)	32 (9.3%)	0.955
DOAK monotherapy	10 (13.2%)	70 (20.3%)	0.857
LMWH/Fondaparinux monotherapy	41 (53.9%)	148 (42.9%)	0.862
dual antiplatelet	7 (9.2%)	37 (10.7%)	0.950
VKA + antiplatelet	1 (1.3%)	8 (2.3%)	0.853
DOAK + antiplatelet	0 (0.0%)	8 (2.3%)	0.630
LMWH + antiplatelet	6 (7.9%)	9 (2.6%)	0.548
triple therapy	0 (0.0%)	3 (0.9%)	0.636
No anticoagulation	0 (0.0%)	8 (2.3%)	0.630
*Comorbidities*			
Ischemic stroke or TIA	23 (30.3%)	103 (29.9%)	0.994
Diabetes mellitus	30 (39.5%)	149 (43.2%)	0.953
Vascular Disease	22 (28.9%)	79 (22.9%)	0.897
Hypertension	73 (96.1%)	326 (94.5%)	0.903
Congestive heart failure	9 (11.8%)	85 (24.6%)	0.773

### Proportion of successful LAA closure (primary efficacy endpoint):

**Fig. 1:**
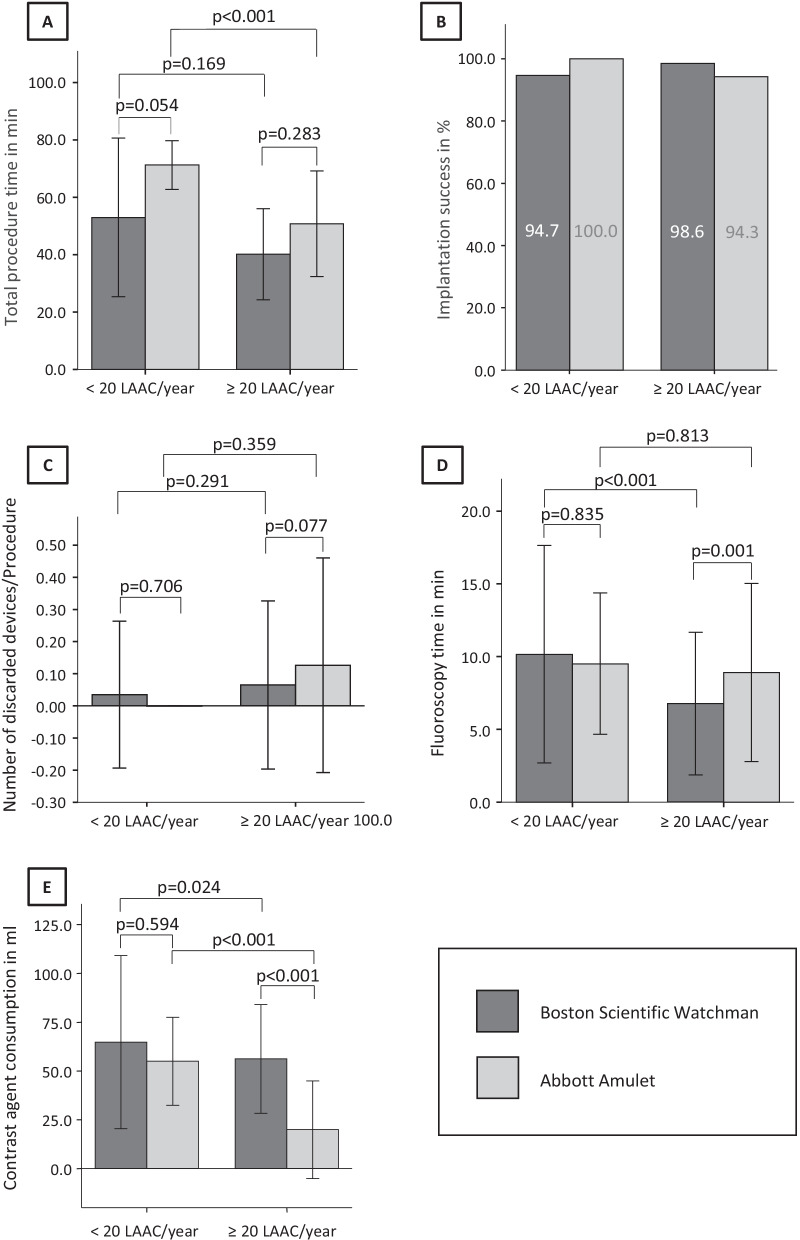
Periprocedural data during LAAC. **A** total procedure time in min; **B** implantation success in %; **C** number of discarded devices/LAAC procedure; **D** fluoroscopy time in minutes; **E** contrast agent consumption in ml. *LAAC/year* left atrial appendage closure procedure/year/study center

The rates of successful LAA closure are illustrated in Fig. 1. Successful implantation of the LAAC device could be performed in a total of 467 (97%), in more than 94% of procedures across both device and clinic types. The kind of device and the frequency of LAAC per study center had no influence on the success rates (χ^2^ = 4.67, df = 2, *p* = 0.10).

### Occurrence of periinterventional complications (primary safety endpoint):

The occurrence of periinterventional complications during LAAC are shown in Table 3 in detail. The overall complication rate was 2.2% in the Amulet group and 2.3% in the Watchman group. The kind of device and the frequency of LAAC per study center had no influence on the complication rates (χ^2^ = 0.77, df = 2, *p* = 0.68).

**Table 3:** Intraprocedural complications

	Amulet	Watchman	*p* value
*Centres with more than 20 procedures per year*			
Intraprocedural groin complication	1 (1.1%)	3 (1.1%)	0.668
Intraprocedural pericardial effusion not requiring surgery	0 (0.0%)	1 (0.4%)	0.760
Intraprocedural pericardial effusion requiring surgery	1 (0.4%)	1 (1.1%)	0.422
Intraprocedural TIA/Stroke	0 (0.0%)	0 (0.0%)	–
Intraprocedural device embolization	0 (0.0%)	0 (0.0%)	–
Intraprocedural death	0 (0.0%)	0 (0.0%)	–
*Centres with less than 20 procedures per year*			
Intraprocedural groin complication	0 (0.00)	1 (0.9%)	0.950
Intraprocedural pericardial effusion not requiring surgery	0 (0.0%)	1 (0.9%)	0.950
Intraprocedural pericardial effusion requiring surgery	0 (0.0%)	1 (0.9%)	0.950
Intraprocedural TIA/Stroke	0 (0.0%)	1 (0.9%)	0.950
Intraprocedural device embolization	0 (0.0%)	0 (0.0%)	–
Intraprocedural death	0 (0.0%)	0 (0.0%)	–

Total procedure time, fluoroscopy time, contrast agent consumption, number of mismeasured discarded devices (secondary efficiency endpoints):

The comparison of the secondary efficiency endpoints between both device groups in dependence of the frequency of LAAC per clinic are illustrated in Fig. 1. The total procedure time was significantly shorter in the clinics performing more than 20 procedures per year independently of the used device.

Additionally, the total procedure time was significantly shorter in the Watchman group in the clinics performing 20 or more procedures per year (Watchman: 40.2 ± 15.9 min vs. Amulet: 50.8 ± 18.4 min; *p* < 0.000). Also, in clinics with less than 20 procedures per year the procedure time in the Watchman group was in trend shorter than in the Amulet patients (Watchman: 53.0 ± 27.7 min vs. Amulet: 71.3 ± 8.5 min; *p* = 0.111). The fluoroscopy time in the Watchman group was significantly higher in clinics with less than 20 procedures per year (< 20 proc/year: 10.2 ± 7.5 min vs. ≥ 20 proc/year: 6.6 ± 4.9 min; *p* < 0.000). The same trend could be shown in the Amulet group (< 20 proc/year: 9.5 ± 4.9 min vs. ≥ 20 proc/year: 8.9 ± 6.1 min; *p* = 0.813). The amount of contrast agent was lower in the trend in clinics performing less than 20 LAAC per year compared to the high-volume clinics. Thereby, in centers performing 20 or more procedures per year during implantation of an Amulet device the contrast agent consumption was significantly lower compared to a Watchman implantation (Watchman: 56.2 ± 27.9 ml vs. Amulet: 19.9 ± 25.0 min; *p* < 0.000). In study centers with less than 20 LAAC per year in trend less devices with not appropriate size were discarded per procedure (< 20 proc/year: Watchman: 0.04 ± 0.23 dev/proc vs. Amulet: 0 ± 0 dev/proc; *p* < 0.706; ≥ 20 proc/year: Watchman: 0.07 ± 0.26 dev/proc vs. Amulet: 0.13 ± 0.33 dev/proc; *p* < 0.077).

In addition, blood loss during the LAAC procedure was determined based on the hemoglobin curve before and after the intervention. Here it could be shown that there was no relevant difference in the hemoglobin drop under VKA or DOACs (decrease of hemoglobin level VKA: − 0.91 ± 0.95 mmol/l vs. DOAC − 0.73 ± 0.62 mmol/l; *p* = 0.397).

**Fig. 2:**
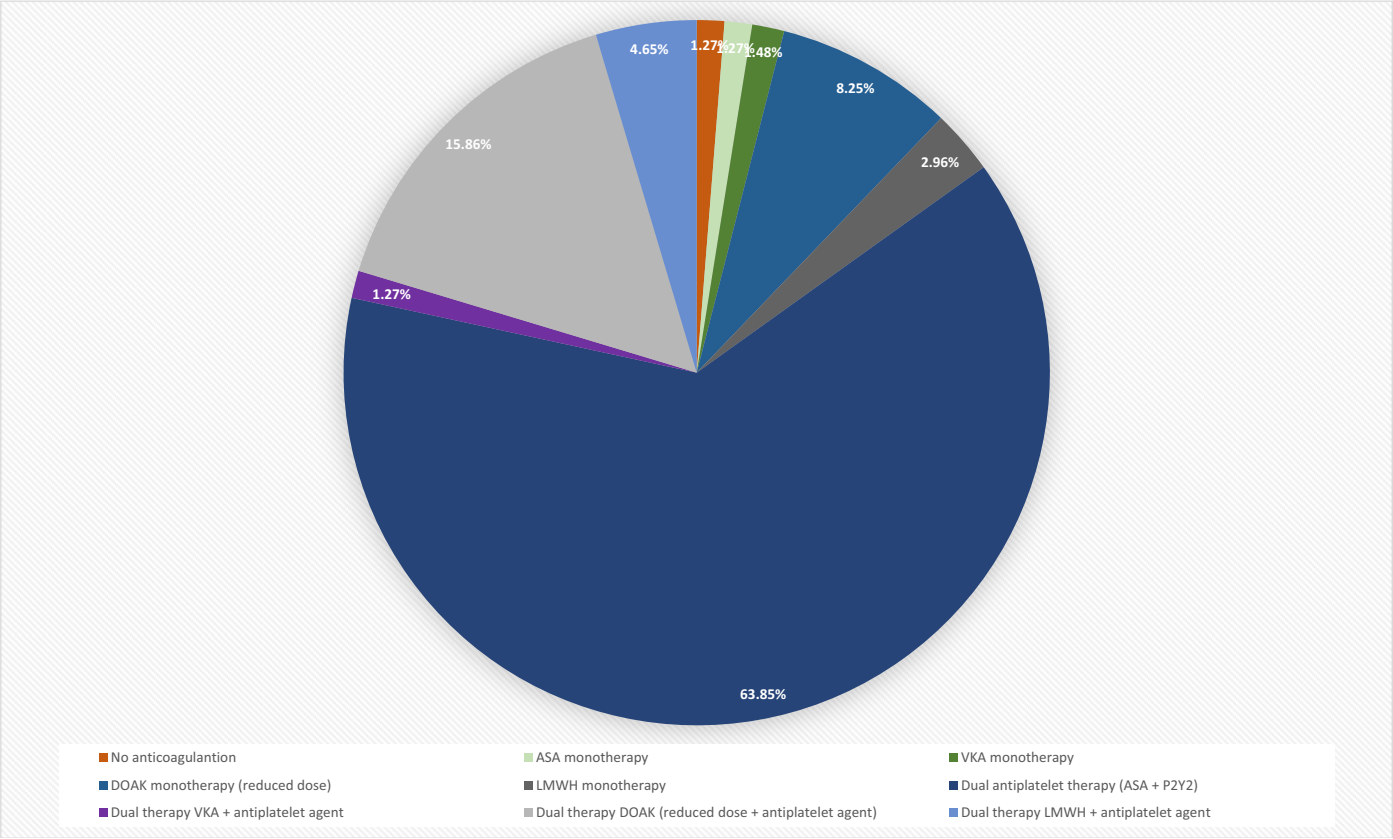
Anticoagulation drug regimens after interventional left atrial appendage closure. *DAPT* dual antiplatelet therapy, *NOAC* novel anticoagulant, *VKA* vitamin K antagonist

The postinterventional antithrombotic therapy is shown in Fig. 2. Thereby 64% of LAAC patients were discharged on dual antiplatelet therapy. Around 16% of patients were discharged on dual therapy containing DOAC in the reduced dose and one antiplatelet.

### Occurrence of device related complications 6 weeks after LAAC (secondary safety endpoint):

The TEE outcome data are summarized in detail in Table 4. In the Watchman group in 4.6% (N = 13) of patients a device related thrombus could be revealed in the TEE. In the Amulet group no thrombus was detected 6 weeks after LAAC (Fisher test, *p* = 0.14). In the Watchman group, 4 patients (1.4%) had a residual device leakage ≥ 5 mm in TEE. No peri-device leak could be revealed in the Amulet group (Fisher test, *p* = 1.00). With respect to the clinical outcome, there were no significant differences in both device groups. In the Watchman group, 10 patients (2.9%) died (non-device related death). There was one patient with a device dislocation in the Watchman group. Additionally, in 0.6% (N = 2) of the patients in the Watchman group suffered from stroke. None of these complications occurred in the Amulet group. There was also no difference in bleeding complications in both device groups.

**Table 4:** Complications follow up in 6 weeks

	Amulet	Watchman	*p* value
Device leck ≥ 5 mm	0	4	0.328
Device associated death	0	0	0.0
Death from other cause	0	10	0.136
TIA or stroke	0	2	0.508
Peripheral thromboembolism	0	0	0.0
Major bleeding	0	1	0.640
Minor bleeding	1	6	0.801
New arrhythmia	0	2	0.508
Device embolisation	0	1	0.640
Other device complications	1	1	0.235

## Discussion

The findings of the present real world ORIGINAL registry support the conclusions of previous randomized trials showing that LAAC can be performed very effectively and safe. Thus, the current registry could demonstrate an implantation success rate of more than 94%. These success rates were achieved independently of the kind of the used device (Watchman or Amulet) and of the LAAC procedure frequency per study center. Further, the overall complication rate was very low with 2.2% in the Amulet group and 2.3% in the Watchman group. In addition, regarding the complication rates, the LAAC procedure frequency per study center has had no influence on the occurrence of intraprocedural complications. But in experience centers with more than 20 LAAC per year the total procedure time, the fluoroscopy time and the amount of used contrast agent was significantly decreased compared to centers with less than 20 LAAC per year. The total procedure time was higher in the Amulet group compared to the Watchman group, but the fluoroscopy time did not differ in either group. The amount of used contrast agent was significantly lower in LAAC with the Amulet compared to the Watchman device. In the current real world setting of the ORIGINAL registry, a wide variety of anticoagulation regimes were registered, whereas the use of a dual antiplatelet medication was the most frequently chosen antithrombotic therapy with 64%. About 22% of patients were discharged under a combination of an anticoagulant (VKA/DOAC/Heparin) and an antiplatelet agent. In terms of device related complications 6 weeks after LAAC there were numerically but not statistically more events in the Watchman group compared to the patients treated with an Amulet device. In 4.5% of Watchman patients a device related thrombus could be detected by TEE compared to 0% in the Amulet group. In 1.4% of the patients in the Watchman group a residual peridevice jet > 5 mm was revealed. In contrast, no residual jet could be found in the Amulet group. No relevant differences in the occurrence of death, thromboembolic events, or bleeding complications were revealed in the clinical follow-up of 6 weeks.

These results are consistent with the conclusions of the previous randomized trials. Thus, this registry data is an important scientific supplement to those from the randomized trials, which could further increase the acceptance of LAAC for stroke prevention in patients with atrial fibrillation, especially in subjects who are not suitable for systemic anticoagulation [3, 4]. In the current ''every day clinical routine''‐registry LAAC was performed in hospitals with a LAAC frequency of less than 20 procedures per year and in centers performing 20 or more LAAC per year. In this context, the perinterventional outcome data of this registry showed no signals, that the efficiency and safety of LAAC is depended on the number of LAACs per center and year. This result should motivate centers to learn the technique of percutaneous LAAC to offer this "local" antithrombotic treatment option to AF patients who are not suitable for systemic anticoagulation, even if it is not an experienced high-volume center. The implantation success rate of 94% in low- and high-volume centers is in line with the data of the PREVAIL trial. In this study about 40% of patients were enrolled in hospitals with new operators. Despite this, an implantation success rate of 95.1% could be achieved. These data confirm that also in the clinical routine the LAAC success rates increased in comparison to the 90.9% procedural success in the initial PROTECT AF trial [3, 4].

The complication rates 6 weeks after LAAC were also similar in the current registry compared to the PREVAIL-Trial. The non-device related death reached 2.9% in the Watchman group in the ORIGINAL registry compared to 2.6% after 18 months in the PREVAIL trial [3]. The rate of thromboembolic complications was also similar in the current registry and the PREVAIL trial [3].

There were minor differences in baseline characteristics between the current registry and the randomized trials. In the PREVAIL trial the mean CHA_2_DS_2_‐VASc Score ranged between 3.8 and 3.9. In contrast, in the current study the mean CHA_2_DS_2_‐VASc Score attained 4.0 in the Watchman and 4.2 in the Amulet group. Considering the aging population with rising co‐morbidities, these data support that LAAC is effective and safe even in patients with higher thromboembolic and bleeding risk.

The EWOLUTION registry data also show that LAAC could be safely performed in patients with a CHA_2_DS_2_‐VASc Score of 4.5 ± 1.6 [5]. Furthermore, the indications for LAAC in the EWOLUTION registry were similar to those of the current study: The in the EWOLUTION registry 73% of the patient have had a history of bleeding [5]. In the ORIGINAL registry patients suffered from previous bleeding complications with 70% in the Amulet group and 81% in the Watchman group. In addition, the choice of the used LAAC device (Boston Scientific Watchman or Abbott Amulet) seems to be equivalent in terms of periprocedural and short-term follow-up complication rates. Supplementary a meta-analysis comparing Watchman and Amplatzer devices for stroke prevention in atrial fibrillation could demonstrate that LAAC devices had low complication rates and low event rates [6]. Efficacy and safety were similar between the systems, except for a higher percentage of insignificant peridevice leakage in the Watchman group [6]. Furthermore, similar safety results could be shown in the recently published prospective randomized Amulet IDE trial [7]. But in terms of the implantation success rates the Amulet IDE trial showed a significantly higher LAA occlusion rate with the Amulet device (98%) compared to the Watchman device (96.8%) [7].

The SWISS APERO Study, which represented the first randomized trial setting side by side the two devices, compared Amulet with Watchman FLX in terms of crossover procedure and residual LAA patency in computed tomography. The clinical outcomes at 45 days did not differ between the two groups, even though not unlike in the IDE trial, the peridevice leakage rate in the transesophageal echocardiography was non-significantly higher [8].

Another important influence for the clinical outcome after LAAC, besides the type of device and the frequency of LAAC procedures per clinic or operator, seems to play the peri- and postinterventional antithrombotic treatment. In this context, a consequence with lower use of postprocedural anticoagulation could be device thrombus, which might be perceived as a risk factor for impending stroke or embolism [9]. But in the EWOLUTION registry in 3.7% of LAAC patients, a device thrombus was detected irrespective to the postprocedural antithrombotic regime [5].

In the ASA Plavix feasibility study with Watchman left atrial appendage closure technology (ASAP-Study) in 6 out of 150 subjects (4%), treated with dual antiplatelet therapy after LAAC, a device thrombus could be revealed [9]. Only one of these six patients was associated with a stroke, whereas in the remaining five cases the device thrombus discovered during surveillance TEEs without clinical sequela [9]. In addition, a single center registry showed a low rate of device thrombi under a antithrombotic treatment for 6 weeks and a subsequent switch to aspirin [10]. Despite these data in the clinical practice, as seen in the current registry, the postprocedural antithrombotic treatment ranges from continuous VKA/DOAC therapy to antiplatelet therapy or no therapy at all. Nevertheless, lower use of anticoagulation does not seem to lead to an increase in device thrombus or stroke. Further, in the large prospective Amplatzer Cardiac Plug registry patients with single LAAC on aspirin monotherapy or no therapy and longer follow-up had fewer cerebral and fewer bleeding events [11]. This finding is encouraging and will be studied further in the ongoing randomized controlled trial in VKA-intolerant patients [12]. Despite all studies, it should be mentioned that the postinterventional antithrombotic treatment should be based on the indication of the left atrial closure procedure. For example, a patient who has received LAAC due to a previous intracranial bleeding should be anticoagulated differently than a patient who suffers from persistent LAA thrombi despite effective anticoagulation.

### Limitations

There are some surmountable limitations of the current registry. First, the study was designed as a registry without randomization of the patients. This could cause a selection bias of the kind of the used LAAC device. Another limitation of this registry is the missing propensity matched analysis between the device groups to rule out potential sampling errors, which would not have been reasonable due to the small sample size. In the ORIGINAL registry only 73% of the patients completed a six week follow up including TEE. In contrast, in the EWOLUTION registry, in 87% of patients a TEE was performed within the first 6 weeks [5]. The small study population of the current registry negatively influences the statistical power regarding the conclusions about death, thromboembolic and bleeding complications. Additionally, the current registry follow-up period of 6 weeks was too short to investigate clinical outcomes of the LAAC patients sufficiently. Moreover, the heterogeneity of the post procedural antithrombotic regimens makes the full assessment of LAAC benefit in this patient population difficult.

## Conclusion

The prospective multicenter ORIGINAL registry demonstrates that LAAC can be performed in everyday clinical routine with a very high procedural success rate. The postprocedural antithrombotic strategy differs widely among the participating study centers. This important issue should be addressed in further randomized trials.

## Data Availability

The datasets generated and analyzed during the current study are available upon reasonable request from the corresponding authors.
